# The Place of Development in the History of Psychology and Cognitive Science

**DOI:** 10.3389/fpsyg.2019.00895

**Published:** 2019-04-24

**Authors:** Gabriella Airenti

**Affiliations:** Department of Psychology, Center for Logic, Language, and Cognition, University of Torino, Turin, Italy

**Keywords:** development, methods, infants, cognitive science, change process

## Abstract

In this article, I analyze how the relationship of developmental psychology with general psychology and cognitive science has unfolded. This historical analysis will provide a background for a critical examination of the present state of the art. I shall argue that the study of human mind is inherently connected with the study of its development. From the beginning of psychology as a discipline, general psychology and developmental psychology have followed parallel and relatively separated paths. This separation between adult and child studies has also persisted with the emergence of cognitive science. The reason is due essentially to methodological problems that have involved not only research methods but also the very object of inquiry. At present, things have evolved in many ways. Psychology and cognitive science have enlarged their scope to include change process and the interaction between mind and environment. On the other hand, the possibility of using experimental methods to study infancy has allowed us to realize the complexity of young humans. These facts have paved the way for new possibilities of convergence, which are eliciting interesting results, despite a number of ongoing problems related to methods.

## Introduction

In this paper, I intend to analyze how the relationship of developmental psychology to general psychology and cognitive science has unfolded. This historical analysis will provide a background for a critical examination of the present state of the art.

Psychology emerged as a scientific discipline with the founding of Wundt’s Laboratory in Leipzig at the end of the nineteenth century (1879)^1^. Wundt’s method, both experimental and introspective, was directed to the study of an adult’s mind and behavior. It is less well-known that only 10 years later, James Baldwin, who had attended Wundt’s seminars in Germany, founded a laboratory of experimental psychology in Toronto in which experiments devoted to the study of mental development were performed. If the occasion that aroused Baldwin’s interest was the birth of his first daughter, actually, “that interest in the problems of genesis–origin, development, evolution–became prominent; the interest which was to show itself in all the subsequent years” (Baldwin, 1930). Baldwin’s work was a source of inspiration for Piaget, certainly one of the most prominent figures in developmental psychology (Morgan and Harris, 2015).

From the origins of psychology as a discipline, general psychology and developmental psychology have followed parallel and relatively separate paths. Two questions are particularly relevant to explain this fact.

From a theoretical point of view, developmental psychology has all along been greatly influenced by biology and evolutionary theory. The founders of developmental psychology have widely analyzed the relation between ontogenesis and phylogenesis (Baldwin, 1895; Piaget, 1928). This analysis resulted in accepting the challenge of explaining development in a broad sense. In his autobiography, Baldwin affirms that already in the 10 years that he spent in Princeton between 1893 and 1903, where he founded another laboratory of experimental psychology, “the new interest in genetic psychology and general biology had become absorbing, and the meagerness of the results of the psychological laboratories (apart from direct work on sensation and movement) was becoming evident everywhere.” Thus, developmental psychology has followed an approach that in general psychology appeared much later^2^.

A second question regards method. Developmental researchers, while manifesting their attachment to experimental procedures, have been confronted with their insufficiency in the study of development. Both for deontological and practical reasons, many aspects of development, in particular in infants and young children, can hardly be investigated experimentally. Thus, a great number of studies in developmental psychology make use of observational methods based on different techniques such as ethnographic methods or parent reports, and the reliability of these methods has been questioned.

This relative separation between studies of adults and children has also persisted with the emergence of cognitive science. Actually, the primary aim of cognitive science, at least at the outset, was to model what we could call an adult static mind. Given a certain output, for instance an action, the task of the psychologist was to reconstruct the inference processes that were at the origin of this same action.

At the beginning of the twenty-first century, psychology and cognitive science have enlarged their scope to include change processes and the interaction between mind and environment, including other minds. Developmental psychology, for its part, has developed nonverbal methods such as looking measures and choice measures that also make it possible to carry out experiments with infants. These facts have paved the way for new possibilities of convergence, which are eliciting interesting results, despite a number of ongoing problems related to methods.

## Psychology, Cognitive Science and Artificial Intelligence

### The Beginning of Cognitive Science

According to the American psychologist George Miller, cognitive science was born on September 11, 1956, the second day of the Second Symposium on Information Theory held at MIT. That day began with a paper read by Allen Newell and Herbert Simon on the state of art of the Logic Theory Machine: a proof on computer of theorem 2.01 of Whitehead and Russell’s *Principia Mathematica*. That very same day ended with the first version of Chomsky’s *The Structures of Syntax*. Miller left the symposium convinced that experimental psychology, theoretical linguistics, and computer simulation of cognitive processes could become parts of a wider whole and that the future of research would be found in the elaboration of this composite whole (reported in Bruner, 1983a). It is Miller who in 1960, together with Eugene Galanter and Karl Pribram, authored a text that may be considered the manifesto of cognitive science and that proclaimed the encompassing of cognitive psychology within the more general framework of information processing (Miller et al., 1960). The assumption was that newly born information science could provide a unifying framework for the study of cognitive systems (Schank and Abelson, 1977).

From a theoretical point of view, the core of this project is the concept of *representation.* Intentional mental states, such as beliefs and perceptions, are defined as relations to mental representations. The semantic properties of mental representations explain intentionality (Pitt, 2017). Representations can be computed and thus constitute the basis for some forms of logic systems. According to the Cognitive Science Committee (1978), which drew up a research project for the Sloan Foundation, all those disciplines, which belong to cognitive science, share the common goal of investigating the representational and computational capacities of the mind and the structural and functional realization of these capacities in the brain.

This point of view constitutes the foundation for what has been called *functionalism* in the philosophy of mind, i.e., the hypothesis that what defines the mind are those features that are independent of its natural realization. The classic functionalist stance is expressed by Pylyshyn in his book on computation and cognition (Pylyshyn, 1984). He maintains that a clear distinction must be made between the functional architecture of the cognitive system and the rules and representations that the system employs.

Functionalism has been greatly discussed and criticized from the beginning (Block, 1978; Dreyfus, 1979). Harnad (1990) identified what has been defined as the *symbol grounding problem*: “How can the semantic interpretation of a formal symbol system be made intrinsic to the system?”

The most exhaustive and most deeply argued critique of functionalism was advanced by Searle, who developed his arguments over time, publishing a number of essays which have given rise to heated debate (Searle, 1980, 1990, 1992). The position taken up by functionalism is that the relationship between the brain and its products, that is to say conscious processes, is mediated by an intermediate level of unconscious rules. This intermediate level is, for functionalists, the level of the program. It is postulated that the rules are computational and that, consequently, the aim of research in cognitive science is to reconstruct these rules. Searle’s objection is that there are only two types of natural phenomena, the brain and the mental states that the brain brings into being and that humans experience. The brain produces mental states due to its specific biological characteristics. When we postulate the existence of unconscious rules, according to Searle, we invent a construct whose aim is to highlight a function, which we believe is especially significant. Such a function is not intrinsic and has no causal power. This argument is particularly interesting because it is founded on the impassable biological nature of the mind. Neither logic nor mathematical or statistical procedures may replace brain as a biological organ.

From another perspective, some scholars have emphasized that functionalism leads to a new form of behaviorism. Putnam (1988) claimed that reducing mental processes exclusively to their functional descriptions is tantamount to describe such processes in behavioristic terms^3^. In psychology, one of the most polemical critics of functionalism as a dangerous vehicle toward a new form of anti-mentalism, which would render vain all the battles waged by cognitivists against classic behaviorism, was a developmental psychologist, Bruner (1990). The centrality of computability as the criterion for the construction of models in cognitive science leads naturally, in Bruner’s opinion, to abandoning “meaning making,” which was the central concern of the “Cognitive Revolution.”

Thus, at least at the outset, cognitive science was devoted to constructing computational models of human inference processes and of the knowledge that is used in performing these inferences. This definition of the object of cognitive science has led at first to designing and implementing problem-solving systems, where the complexity was located in the inference mechanisms, supposed to be the same for all problems (Newell and Simon, 1972). Later, systems were implemented where reasoning was associated with specific and articulated knowledge representation (Levesque and Brachman, 1985).

Notably, the aspect that was absent from this view of cognitive science was learning. This lack, according to Gentner (2010), could be partly explained as a reaction to behaviorism, which was completely centered on learning. In fact, there were also philosophical reasons. Chomsky and Fodor, who were among the most influential members of the cognitive science community, were highly critical of the concept of learning. In their view, learning as a general mechanism does not exist, and Fodor even went so far as to state explicitly that no theory of development exists either (Fodor, 1985).

Thus, cognitive science was born essentially as a reaction to behaviorism and took its legitimacy from the use of methodologies developed within artificial intelligence. These methodologies were supposed to make explicit how mental representations produced human activity in specific domains. However, this approach had a price: it separated the mind from its biological basis and from the context in which human activity takes place. There was no place for development, interaction, and variation due to biological or social causes^4^. This theoretical choice explains Bruner’s disillusion. For Bruner, cognitive science had fallen back into the behaviorism against which it originated, and no interesting relation could be established with developmental psychology. Developmental psychology is founded on the premise that a human being develops in interaction with the physical world and the society of other humans.

### Cognitive Science in the Twenty-First Century

Cognitive science has changed considerably from its beginning. An obvious novelty concerns the increased importance assumed by learning with the emergence of connectionism (Hinton, 1989).

When connectionist models were introduced, there was much debate regarding the relation of neural networks with the functioning of the human brain and their ability to address higher forms of thought (Fodor and Pylyshyn, 1988; Quinlan, 1991; Chalmers, 1993). Later, philosophical discussion was replaced by empirical considerations. Networks are an efficient computational tool in some domains and are often used jointly with symbolic computations (Wermter and Sun, 2000). Moreover, in recent advancements of artificial Intelligence, neural networks have been largely replaced by a variety of techniques of statistical learning (Forbus, 2010).

More interesting for my purpose is the changes that the general philosophy of cognitive science has undergone due to the problems that have emerged with classic symbolic models. At its origin, the core of cognitive science was the relation between psychology and artificial intelligence. In the original project, this marriage was to be fruitful for both disciplines. Artificial intelligence expected from psychology the analysis of high-level mental mechanisms that, once simulated on a computer, could improve the efficiency of artificial systems. With computer simulation, psychology was to acquire a method to validate its models. However, this marriage, which for a while has been very productive and has generated many interesting ideas, ultimately failed. Artificial intelligence has evolved computing techniques that produce efficient systems without asking anymore if these techniques replicate human mental processes more or less faithfully. In psychology, the constraint to produce computational models has again restricted its scope (Airenti and Colombetti, 1991).

Thus, the results of cognitive science of the twentieth century have led to a shift in cognitive science that has emerged with this century. Some researchers have proclaimed that the theoretical hypothesis that minds functionalities can be modeled disregarding the fact that they operate on the external world through the body could no longer be accepted. This new approach implies accounting for the biology of the mind/body unity and the interaction with the external world, both physical and social. One source of inspiration for this new turn came from Varela et al. (1991), who proposed the concept of the *embodied mind*. Actually, the concept of embodiment includes many rather disparate inspirations, from Merleau-Ponty and phenomenology to Buddhism. I do not analyze these questions here. What interests me is the mere assumption that cognition is grounded in the world.

This new turn corresponds to the major importance assumed by robotics. It might be exaggerated to say that the role played by artificial intelligence in the past is now assumed by robotics. However, it is clear that the aim of constructing artificial actors that interact with the world and/or with humans has again established a link between the study of humans and the production of artificial systems. With respect to the past, the focus is no longer on the symbolic function of the mind, but on the mind embedded into a physical device that interacts with the external world. This evolution is linked to the enlarged scope of present robotics that goes well beyond traditional tasks such as farm automation. The ambition is to construct robots that may cooperate with humans in a multiplicity of tasks, including, for instance, assisting aged or disabled people or interacting with autistic children. Social robotics has then evolved toward biologically inspired systems, based on the notions of self-organization and embodiment (Pfeifer et al., 2007). This new development has led to question once again psychologists about those characteristics that make humans what they are. If robots must be able to interact with humans, they should show those same characteristics (Kahn et al., 2007). Can robots be endowed with intentionality, emotions, and possibly empathy?

Here, again a functionalist position appears. For some authors, the fact that the robot’s internal mechanisms are grounded in physical interactions with the external environment means that they truly have the potentiality of intrinsic intentionality (Zlatev, 2001). This means, for them, that a mind is embodied in a robot. To the question of whether robots can have emotions, Arbib and Fellous (2004) answer that a better knowledge of biological systems will allow us in the future to single out “brain operating principles” independent of the physical medium in which they are implemented. This new form of functionalism is currently contrasted with an approach that considers that mental states and emotions are not intrinsic but can only be attributed to robots by humans (Ziemke et al., 2015). Robots’ embodiment does not overcome the objection that was addressed to traditional artificial intelligence, namely that mental states and emotions can only be produced by a biological brain (Ziemke, 2008). This latter position maintains that the relevant question for human-robot interaction is not that robots must be intentional beings, but that they must be perceived as such by humans (Airenti, 2015; Wiese et al., 2017).

In conclusion, we can say that cognitive science was born as a way to renew psychology through a privileged connection with artificial intelligence. In the present state of research, it is social robotics that is attempting to establish a connection with biological sciences, psychology, and neuroscience, in order to build into robots those functionalities that should allow them to successfully interact with the external physical and social world. However, the main fundamental philosophical problems remain unchanged. One could still argue, as Searle did, that human mentality is an emergent feature of biological brains and no logical, mathematical or statistical procedure can produce it.

### Present Questions for Cognitive Science

The question that we may raise today is this: what is cognitive science for? The relation that psychology has established with the sciences of the artificial has hidden the fact that a number of phenomena, which are essential for explaining the functioning of the human mind, have been largely ignored. This failure in explanation, which has concerned, for instance, the managing of mental states and emotions, and many complex communicative phenomena, is fundamentally linked to the fact that the mind is constantly in interaction with the physical and social world in a process of development. The primitive idea of cognitive science was to go beyond traditional psychology to enrich the study of mind with the contributions of other disciplines that also investigated human mind, such as linguistics, philosophy, and anthropology. This approach, which concerns the definition of the field of cognitive science, has been quite early reinterpreted as a problem of formalism. The question posed has been: how could psychology produce scientific models of human thought? Hence, the importance assumed by computer modeling as a means of replacing more traditional logical, mathematical, and statistical models. However, this theoretical choice has generated a major ambiguity, because computer models that are founded on logical, mathematical, or statistical formalisms have been seen as possibly equivalent to the mind. Once the fallacy of this equivalence appears—because no artificial model may replace the causal power of the human brain—we are left with some formal models with very limited psychological significance. What has been lost is the richness that cognitive science was supposed to acquire by connecting different disciplines. In particular, for many years, this approach has prevented general psychology from connecting with developmental psychology, a field of studies that, since Baldwin, had already posed the problem of the construction of the human mind as the result of biological development and social interaction.

## The Study of Development

### Biology and Development in the Debate Between Piaget and Chomsky

Studying development necessarily implies considering the fact that humans are biological systems that are certainly particularly complex but also share many characteristics with other living beings. Thus, in the field of developmental psychology, many questions have emerged concerning the link between development and evolution, the relation between genetic endowment and the influence on acquisition of environment (a concept that includes physical environment, parenting, social rules, etc.), and the nature of learning.

For Piaget, who came to developmental psychology from natural sciences, development had to be seen in the light of the theories of evolution. Intelligence, for him, is a particular case of biological adaptation, and knowledge is not a state but a process. Through action, children explore space and objects in the external world, and in this way, for instance, they learn the properties of the objects and their relations. These ideas, which sound rather contemporary to us, were considered as problematic in the past and prevented the establishment of a relationship between the study of development and the study of cognition in general. It is only in this century that development has been integrated into evolution studies *via* the so-called *evo-devo* approach and that these ideas have given rise to an interest in psychology (Burman, 2013).

Actually, some aspects of Piaget’s perspective were problematic. Piaget supported his theory using what was considered a Lamarckian vision of evolution that assumed the inheritance of acquired characteristics. He had a well-known debate at the end of his life (1975) with Noam Chomsky on language acquisition, and outstanding biologists who also participated to the debate contested the validity of his use of the concept of *phenocopy* (Piattelli-Palmarini, 1979/1980). In fact, on this point, Piaget had been influenced by Baldwin, who proposed what is known as *Baldwin’s effect* (Simpson, 1953). This effect manifests in three stages: (1) Individual organisms interact with the environment in such a way as to produce nonhereditary adaptations; (2) genetic factors producing similar traits occur in the population; and (3) these factors increase in frequency under natural selection (taken from Waddington, 1953). Later, Piaget revised his own theory and updated Baldwin’s effect under the influence of Waddington (Burman, 2013). Recently, epigenetic theories have emerged in biology, and the importance of development is generally accepted. On the developmental side, it has been proposed that Piaget’s theory might be replaced as a metatheory for cognitive development by evolutionary psychology (Bjorklund, 2018).

The debate between Chomsky and Piaget is interesting because it is a clear example of the impossibility of dialogue between one of the fathers of cognitive science and the scholar who, at that moment, personified developmental psychology. Piaget was unable to justify his position that grammar rules could also be accounted for by sensorimotor schemata, and Chomsky appeared to have won the debate. At the same time, Chomsky presented the emergence of syntactic rules in the child’s mind, excluding in principle any possible form of learning. However, in hindsight, we know how the task of establishing abstract principles of universal grammar proved to be arduous, underwent many substantial changes and is not yet realized.

Another controversial aspect of Piaget’s position was his adherence to the recapitulation theory, i.e., the idea originally proposed by Haeckel, that ontogeny recapitulates phylogeny. It is this principle that motivated Piaget’s study of development as a way of contributing to the study of the evolution of human thought (Koops, 2015). However, this position has as its consequence the idea that primitive populations would exist wherein we might find adult thought processes that in modern civilizations are typical of young children.

What is striking in this debate is that the specific biological model that Piaget adopted was not the only point of disagreement. What was questioned was in general the relevance of development for the study of a basic human ability such as language. Certainly, in the work of the first figures of developmental psychology, we find a baffling mix of very interesting ideas regarding the place of humans as biological entities in evolution and a difficulty in taking into account the complexities of actual biological theories and of social aspects such as cultural variation. At the same time, these scholars were confronted with objections from cognitive scientists who did not admit the relevance of investigating development for the study of the human mind.

### The Interactionist Perspective

Piaget’s perspective was, in a sense, paradoxical. This perspective considered children’s development as the product of their action on the environment, but at the same time postulated a rather rigid succession of stages that led to adult thought and excluded the importance of the social aspects of this environment in the first years. In fact, infants and young children were considered closed in their egocentrism and unable to take advantage of their interactions with adults and peers.

These aspects have been criticized within developmental psychology, where a cultural turn, fathered by Vygotsky (1962/1986) and mainly interpreted in the United States by Bruner (1990), has arisen. For both these authors, biological factors are considered an endowment of potentialities that develop in a society of co-specifics and are submitted to variability and to cultural variation.

Bruner was, at the outset, an enthusiastic supporter of cognitive science and in particular of the mentalist theory of language proposed by Chomsky (Bruner, 1983b). Later, however, the primacy that Chomsky assigns to syntax turned out to be unsatisfactory to Bruner, according to whom language is fundamentally a communicative device. The problem of language acquisition is thus redefined as the development of a communicative capacity that appears in the prelinguistic stage. This position was the result of Bruner’s work on preverbal communication carried out at the Center for Cognitive Studies at Harvard University starting in 1966.

For Bruner, language requires the maturation of cognitive structures, which underlie intentional action in general. His debt to Piaget with regard to the importance of action is evident. Language is “a specialized and conventionalized extension of cooperative action” (Bruner, 1975). In this, he rejoins the communication theories proposed within the philosophy of language by Austin (1962) and Grice (1989).

Bruner’s studies are part of a revolution in developmental studies in which more careful scrutiny and more sophisticated experimentation led to the discovery that children begin to engage in rather complex cognitive activity very early on. Prior to these studies, many of the aspects relating to infant cognition were not taken into consideration. The prejudice that saw human development as the slow acquisition of rationality prevented researchers from seeking elements of complexity in the cognition of a new-born.

In brief, since its origin, developmental psychology has undergone an important change. At the outset, the idea was that what characterized human cognition was adult rational thought, and studying development meant understanding the stages that led to this achievement. Later, the goal became understanding the development of the different faculties that characterize cognition starting from birth. This goal has also opened the door to comparative studies.

### The Problems of Method

Developmental psychologists have always struggled with problems of method.

Piaget frequently discussed his observations of his three children. Studies on language acquisition have often benefited from researchers’ observations of their own children (see, for instance, Stern and Stern, 1928). These procedures, which have been considered as barely scientific by other psychologists, have provided useful inspiration for further research. Note that Darwin’s observations of his children were a fundamental source for his work on emotions (Darwin, 1872/1965).

Ethical reasons forbid experiments, which may perturb children. Moreover, conceiving experiments that have ecological validity is even more difficult to do with young children than with adults. Hence, the necessity of using different methods in order to produce data that cannot be collected using classic experimental procedures. Without using observational methods, for instance, it is not possible to assess the spontaneous appearance of a given phenomenon (Airenti, 2016). Furthermore, some behaviors may appear only in specific situations and would go unnoticed if they were not observed by caregivers who may see children at different moments of the day and in different situations. Thus, developmental psychologists have used different methodologies, classic experiments but also fieldwork, ethological observation, and parent reports.

A fundamental advancement was the development of techniques permitting to assess infants’ and young children’s abilities in experiments. A key element was the elaboration of the habituation paradigm (Fantz, 1964; Bornstein, 1985). After repeated exposure to a stimulus, infants’ looking time decreases due to habituation and increases when a novel stimulus is presented. Habituation allows us to understand if infants discriminate among different stimuli.

In particular for language studies, nonnutritive sucking (Siqueland and De Lucia, 1969) has been used. This is an experimental method based on operant conditioning allowing one to test infants’ discrimination of and preference for different stimuli. This technique has been used to show, for instance, that infants already acquire in the mother’s womb the ability to recognize and prefer the prosody of a language and of familiar voices (DeCasper and Fifer, 1980).

Currently, the most utilized technique with infants is preferential looking or reaching. In this technique, two stimuli are presented together and what is measured is the infant’s preference. Specific types of this technique are used to claim surprise, anticipation, and preferences for novel or familiar stimuli and to evaluate preference over and above novelty or familiarity (Hamlin, 2014)^5^.

Another technique presently used to investigate infant cognitive development is EEG recordings, even if creating infant-friendly laboratory environments, age-appropriate stimuli, and infant- friendly paradigms requires special care (Hoehl and Wahl, 2012).

The development of these experimental techniques has vastly enlarged the scope of infant studies. In particular, a new research trend has emerged aimed at discovering what has been called the core knowledge (Spelke, 2000; Spelke and Kinzler, 2007). The idea is that at the basis of human cognition, there is a set of competencies, such as representing objects, action, number and space, which are already present in infants and which underlie and constrain later acquisitions. Researchers have also been working on other possible basic competencies such as social cognition (Baillargeon et al., 2016) and morality (Wynn and Bloom, 2014).

In the literature, debate continues surrounding the replicability and robustness of findings obtained within these experimental paradigms, in particular with respect to infants’ and toddlers’ implicit false belief and morality (Hamlin, 2014; Tafreshi et al., 2014; Baillargeon et al., 2018; Sabbagh and Paulus, 2018).

This debate also involves the relation between development and evolution. For Tafreshi and colleagues, for instance, the idea of core knowledge would involve a consideration of high-level cognitive capacities as biologically predetermined instead of constructed in interaction with the environment. This is not the perspective of those who consider that development does exist in the social environment but is constrained by a number of basic competencies (Hamlin, 2014). An important element of this perspective is comparing human and animal capacities. In fact, research has shown that such basic competencies also exist in some form in animals. For instance, numerous studies have shown that adult nonhuman primates have the core systems of object, number, agent representations, etc. (Spelke and Kinzler, 2007).

These preoccupations have also informed work by Tomasello and the Leipzig group. “All we can claim to have done so far–writes Tomasello–is to establish some comparative facts–organized by some theoretical speculations–that hopefully get us started in the right direction toward an evolutionary informed account of the ontogeny of uniquely human psychology” (Tomasello, 2018). Comparing experimental work on great apes and young children has led him to formulate the hypothesis that the factors marking the difference between these two groups are different aspects of social cognition. Nonhuman primates have some basic capacities in these areas. In humans, the evolved capacity for shared intentionality transforms them in the species-unique human cognition and sociality (Tomasello and Herrmann, 2010).

Tomasello’s work has also aroused criticism. In this case, the criticism is because his research, both with young children and primates, uses experimental methods and is carried out in a laboratory. Fieldwork primatologists have claimed that primates in captivity, tested by someone of another species, cannot display the abilities that their conspecifics display in their natural environment (Boesch, 2007; De Waal et al., 2008). Tomasello answered this criticism by maintaining that the fact of being raised in a human environment enhances primates’ capacities (Tomasello et al., 1993; Tomasello and Call, 2008).

In conclusion, in developmental psychology, a multiplicity of methods has been applied, and the debate over their respective validity and correct application continues. However, what is not in question is that development is a complex and multifaceted phenomenon that must be analyzed as such and from different points of view.

A paradigmatic case in the present research is the study of the theory of mind. Discovering how subjects represent their own mind and other minds was proposed in 1978 by Premack and Woodruff as a problem of research on primates, and in a short time, it has become one of the main topics in developmental research (Premack and Woodruff, 1978). It is currently being studied in groups of different ages, from infants to the elderly, both in typical and clinical subjects and using different methodologies, from classic experiments to clinical observation. Moreover, a number of studies investigate individual and cross-cultural variation and its role in human-robots interactions. Philosophers have contributed to the definition of this phenomenon, and neuroscientists are working to discover its neural basis.

### Computational Models of Development

Some researchers have pursued the goal of constructing computational models of cognitive development using different computational approaches (for a review, see Mareschal, 2010). However, as the author of this review remarks, all the models have explored cognition “as an isolated phenomenon”, i.e., they did not consider the physical and social context in which development unfolds.

Karmiloff-Smith, a developmental psychologist who proposed the most interesting theory about developmental change as an alternative to Piaget’s, considered that a number of features of her RR (*representational redescription*) model happened to map onto features of connectionist models (Karmiloff-Smith, 1992; for a review of these models, see Plunkett et al., 1997). However, she also remarks that connectionist models have modeled tasks, while development is not simply task-specific learning, as it involves deriving and using previously acquired knowledge^6^.

One result of the dissatisfaction with the results deriving from the relation between cognitive psychology and artificial intelligence and the concomitant increase in interest in embodied cognition has been the growth of *developmental robotics* (Lungarella et al., 2003). The aim of this field is to produce baby robots endowed with sensorimotor and cognitive abilities inspired by child psychology and to model developmental changes (Cangelosi and Schlesinger, 2018). This approach has led to the comparison of results in experiments with robots and children. This is a promising field, even if it does not overcome the problems described above regarding the specificity of tasks that does not allow to account for infants’ ability to utilize previously differently acquired knowledge in the performance of a given task.

In conclusion, some approaches within cognitive science have acknowledged the usefulness of studying children in order to understand the mechanisms of development. Especially in the case of developmental robotics, this has allowed for studying the interaction of different capacities such as sensorimotor abilities, perception, and language. At the same time, the computational constraints do not allow for overcoming task specificity.

## Concluding Remarks

I have argued that since their beginning, general psychology and developmental psychology have followed parallel paths that have only occasionally converged. The reason is due essentially to methodological problems that have involved not only research methods but also the very object of inquiry.

Psychology was founded with the ambition of becoming a science performed in laboratories and based on experimental work. However, as early as in 1934, Vygotsky had already deplored the attempt to achieve scientific standards by limiting the importance of general issues. “As long as we lack a generally accepted system incorporating all available psychological knowledge, any important factual discovery inevitably leads to the creation of a new theory to fit the newly observed facts” (Vygotsky, 1962/1986, p. 13).

The birth of cognitive science has taken important steps toward constructing links with other disciplines and also other ways to study cognition. However, this opening was soon transformed in the search for a unifying methodology, namely computer modeling, as a guarantee of scientific results. Many interesting ideas have been generated. However, after four decades of work in this direction, it has become impossible to ignore that too many important aspects of the human mind and activity have been eluded.

The relative isolation of developmental psychology came from the prejudice, also shared by eminent developmental psychologists like Piaget, that what characterizes human cognition are adult cognitive abilities.

However, from the start, developmental psychology was not limited to investiganting the specificity of children’s cognition. It devoted attention to what makes development possible, including biological endowment and cultural transmission; whether an infant should be considered a blank slate or if one can define some pre-existent basic abilities; what makes humans different from animals and nonhuman primates; and how specific human abilities such as language have evolved.

At present, a rapprochement between adult and child studies is made possible by different factors. The possibility of using experimental methods to study infancy has allowed us to realize the complexity of young humans. Moreover, development is increasingly being considered as a phenomenon not only characterizing childhood but also present over the life span, including both the acquisition and the decay of mental abilities (Bialystok and Craik, 2006). Studying the human mind means studying how the human mind changes in interaction with the external environment all life long. In this sense, the study of human mind is inherently connected with the study of its development.

An important question of method emerges here. We have observed that over the years, developmental psychologists have sought to construct methods that can be reliable and at the same time can adequately address the topics under discussion here. The achievement of finding ways to carry out experiments with infants and nonhuman primates has been an important advancement in this perspective. This advancement has garnered both praise and criticism. To be reliable, experiments with infants require very rigorous procedures. Frequently, a detailed analysis of procedures is necessary to explain divergent results. However, it can be noted that reproducibility is an open problem for psychological science in general (Open Science Collaboration, 2015). For nonhuman primates, the ecological validity of laboratory experiments has been questioned. More generally, it has been shown that in the field of developmental psychology, experimental studies do not completely replace other methodologies, but rather should coexist with them.

The human mind is complex, and all the methods that have been proposed in different disciplines may be useful in advancing our knowledge of it. The explanation of this complexity was the main goal underlying the proposal of cognitive science and is the perspective we must pursue in the future.

On this ground, the paths of psychology and developmental psychology may reconverge.

## Author Contributions

The author confirms being the sole contributor of this work and has approved it for publication.

### Conflict of Interest Statement

The author declares that the research was conducted in the absence of any commercial or financial relationships that could be construed as a potential conflict of interest.

The reviewer MT declared a shared affiliation, with no collaboration, with the author to the handling editor at time of review.

## Footnotes

^1^The very earliest date was 1875 and that same year William James’ laboratory at Harvard in the United States was established (Harper, 1950).

^2^William James was influenced by Darwin and this appears in particular in his conceiving the mind as a function and not as a thing (Bredo, 1998). However, his book *The Principles of Psychology*, first published in 1890 and later revised several times, ignored child development. In the chapter devoted to methods and snares in psychology, he adds to introspective observation and experimental method the comparative method. “So it has come to pass that instincts of animals are ransacked to throw light on our own; and that the reasoning faculties of bees and ants, the minds of savages, infants, madmen, idiots, the deaf and blind, criminals, and eccentrics, are invoked in support of this or that special theory about some part of our own mental life” (James, 1983, p. 193). If he admits that “information grows and results emerge”, he also cautions that “there are great sources of error in the comparative method” and that “comparative observation, to be definite, must usually be made to test some pre-existing hypothesis” (James, 1983).

^3^Putnam was actually the first to employ the term *functionalism*, and his aim in doing so was anti-reductionist. In his 1975 work he used the comparison with a computer program to show that psychological properties do not have a physical and chemical nature, even though they are realized by physical and chemical properties (Putnam, 1975).

^4^Hewitt (1991) highlights the difficulties inherent in constructing artificial systems, which, like social systems, are founded on concepts such as commitment, cooperation, conflict, negotiation, and so forth.

^5^Gaze and eye-tracking techniques are normally used in psychological research with adults (Mele and Federici, 2012) but it is in developmental studies that they have had a dramatic impact on the possibilities of inquiry.

^6^A different approach that has given origin to formal models and simulations is the paradigm that views the developmental process as a change within a complex dynamic system. Cognition in this perspective is embodied in the processes of perception and action (Smith and Thelen, 2003).
